# Medicaid Expansion and Mortality Among Patients With Breast, Lung, and Colorectal Cancer

**DOI:** 10.1001/jamanetworkopen.2020.24366

**Published:** 2020-11-05

**Authors:** Miranda B. Lam, Jessica Phelan, E. John Orav, Ashish K. Jha, Nancy L. Keating

**Affiliations:** 1 Department of Health Policy and Management, Harvard T.H. Chan School of Public Health, Boston, Massachusetts; 2 Department of Radiation Oncology, Brigham and Women’s Hospital, Dana-Farber Cancer Institute, Boston, Massachusetts; 3 Division of General Internal Medicine, Department of Medicine, Brigham and Women’s Hospital, Boston, Massachusetts; 4 Department of Biostatistics, Harvard T.H. Chan School of Public Health, Boston, Massachusetts; 5 School of Public Health, Brown University, Providence, Rhode Island; 6 Department of Health Care Policy, Harvard Medical School, Boston, Massachusetts

## Abstract

**Importance:**

Medicaid expansion under the Patient Protection and Affordable Care Act may be associated with increased screening and may improve access to earlier treatment for cancer, but its association with mortality for patients with cancer is uncertain.

**Objective:**

To determine whether Medicaid expansion is associated with improved mortality among patients with cancer.

**Design, Setting, and Participants:**

This is a quasi-experimental, difference-in-difference (DID), cross-sectional, population-based study. Patients in the National Cancer Database with breast, lung, or colorectal cancer newly diagnosed from January 1, 2012, to December 31, 2015, were included. Data analysis was performed from January to May 2020.

**Exposure:**

Living in a state where Medicaid was expanded vs a nonexpansion state.

**Main Outcomes and Measures:**

The main outcome was mortality rate according to whether the patient lived in a state where Medicaid was expanded.

**Results:**

A total of 523 802 patients (385 739 women [73.6%]; mean [SD] age, 54.8 [6.5] years) had a new diagnosis of invasive breast (273 272 patients [52.2%]), colorectal (111 720 patients [21.3%]), or lung (138 810 patients [26.5%]) cancer; 289 330 patients (55.2%) lived in Medicaid expansion states, and 234 472 patients (44.8%) lived in nonexpansion states. After Medicaid expansion, mortality significantly decreased in expansion states (hazard ratio [HR], 0.98; 95% CI, 0.97-0.99; *P* = .008) but not in nonexpansion states (HR, 1.01; 95% CI, 0.99-1.02; *P* = .43), resulting in a significant DID (HR, 1.03; 95% CI, 1.01-1.05; *P* = .01). This difference was seen primarily in patients with nonmetastatic cancer (stages I-III). After adjusting for cancer stage, the mortality improvement in expansion states from the periods before and after expansion was no longer evident (HR, 1.00; 95% CI, 0.98-1.02; *P* = .94), nor was the difference between expansion vs nonexpansion states (DID HR, 1.00; 95% CI, 0.98-1.02; *P* = .84).

**Conclusions and Relevance:**

Among patients with newly diagnosed breast, colorectal, and lung cancer, Medicaid expansion was associated with a decreased hazard of mortality in the postexpansion period, which was mediated by earlier stage of diagnosis.

## Key Points

**Question:**

Is Medicaid expansion associated with changes in mortality for patients with cancer?

**Findings:**

In this cross-sectional study of 523 802 patients with newly diagnosed breast, colorectal, or lung cancer, Medicaid expansion was associated with decreased mortality in expansion states compared with control nonexpansion states. This mortality improvement appeared to be mediated by earlier stage of cancer at diagnosis.

**Meaning:**

In this study, Medicaid expansion was associated with a decreased hazard of mortality among patients with newly diagnosed breast, colorectal, and lung cancer.

## Teaser

This cross-sectional study examines whether Medicaid expansion is associated with improved mortality among patients with breast, colorectal, and lung cancer.

## Introduction

The Patient Protection and Affordable Care Act expanded Medicaid eligibility to nonelderly adults with incomes at or below 138% of the federal poverty level in participating states. By March 2020, 36 states and Washington, DC, had expanded Medicaid,^1^ with more than 20 million US residents obtaining coverage.^2^

Previous studies among patients with cancer have shown Medicaid expansion to be associated with fewer patients being uninsured, increased screening,^3^ and earlier stage of diagnosis,^4,5,6,7,8,9,10,11^ with mixed results on racial and socioeconomic disparities.^12,13,14,15^ Studies have shown increased colorectal cancer screening among low-income and Black patients within early expansion states.^13^ Medicaid expansion is also associated with reduced racial disparities in access to high-volume hospitals for cancer surgery and increased rates of cancer surgery among low-income patients. However, it is also associated with increased racial disparity in access to high-quality hospitals for cancer surgery.^12,15^ The association of Medicaid expansion with mortality for patients with cancer remains unknown. Medicaid expansion might improve mortality through earlier detection, earlier stage of diagnosis, and/or improved access to recommended treatments. Alternatively, expansion could worsen mortality by overloading the oncology workforce and hospitals^16,17^ with an influx of newly covered patients, leading to increased wait times seen in other settings.^18,19^ As nonexpansion states engage in ongoing discourse about whether to expand Medicaid, empirical data would be helpful in guiding their decisions.

In this study, we sought to build on the data showing that Medicaid expansion increased detection of early-stage cancer by examining 3 questions. First, did mortality for patients with cancer change in states that expanded Medicaid compared with states that did not expand Medicaid? Second, if there were changes, could detection at earlier stage be mediating those changes? Finally, did similar mortality changes occur among at-risk populations (ie, patients living in lowest income neighborhoods and Black vs White patients)?

## Methods

### Data Source

Data were obtained from the National Cancer Database (NCDB), which contains patient-level data on more than 70% of new cancer diagnoses from approximately 30% of US hospitals. The Harvard University institutional review board approved this study and waived the need for informed consent because the data are publicly available and deidentified. This study follows the Strengthening the Reporting of Observational Studies in Epidemiology (STROBE) reporting guideline.^20^

### Patient Population

We studied patients with newly diagnosed invasive breast, colorectal, and lung cancer from January 1, 2012, to December 31, 2015. We only included patients for whom this was their first cancer diagnosis (eAppendix 1 in the Supplement). These cancers were selected because they are common, amenable to screening, and treated for cure in the nonmetastatic setting. Outcomes for these patients could be sensitive to changes in insurance and access to care. A recent study^9^ found that expansion states had a higher rate of patients whose cancer was diagnosed at an earlier stage compared with nonexpansion states after Medicaid expansion for these cancers. NCDB provides a variable indicating the expansion status of each patient’s state of residence. We classified expansion states as early (ie, before the Patient Protection and Affordable Care Act) and as January 2014 (eTable 1 in the Supplement) to compare with the nonexpansion group. We excluded patients in late expansion states (mid-2014 to 2018 expansion) because we could not determine the time of expansion without additional information (state of residence) and had insufficient postexpansion data. We also excluded patients for whom NCDB did not report expansion status (missing or aged <40 years). We excluded patients with missing data on insurance status or cancer stage (26 819 patients [4.9%]). eFigure 1 in the Supplement depicts the population flowchart.

### Outcomes

The primary outcome was mortality, which is defined in the NCDB as months from initial cancer diagnosis to death. Information on cancer-specific mortality is not available in NCDB. Twenty-four states and the District of Columbia expanded their Medicaid programs on January 1, 2014, which marked the beginning of the postexpansion period in our primary analysis (eTable 1 in the Supplement). Early expansion states formally expanded on January 1, 2014, but a smaller subset could join Medicaid earlier through a waiver process. We included these states in our analyses, understanding that they could bias our results toward the null.

### Statistical Analysis

We first summarized baseline characteristics of our cohort in expansion (early and January 2014) and nonexpansion states. Missing data were presented for zip code–level variables and were included as a separate category. Our primary Cox proportional hazards model compared the change in survival times—from the periods before to after expansion—between patients in expansion and nonexpansion states. The outcome, survival, was available for patients whose cancer was diagnosed by December 31, 2015. The primary independent variables in the model were expansion status (expansion vs nonexpansion), period (before expansion [2012-2013] vs after expansion [2014-2015]), and the interaction between expansion status and period. Significance of the difference-in-difference (DID) interaction term indicates that the change in survival over time in expansion states was different from the change in survival over time in nonexpansion states.

Our primary model and corresponding adjusted Kaplan-Meier curves were adjusted for age, sex, cancer type, urban location, Charlson Comorbidity Index score, and the interaction between sex and cancer type. Variables were categorized as shown in Table 1. We did not adjust for cancer stage or other patient characteristics (education, income, insurance, and race) that could have acted as mediators of Medicaid expansion. Race categories were defined per the NCDB (eAppendix 2 in the Supplement). To determine the role of intermediary variables, our second model adjusted for education, income, insurance, and race. Our final model also adjusted for stage.

**Table 1.** Baseline Patient Characteristics

Characteristic	Patients, No. (%)	
	Nonexpansion states (n = 234 472)	Combined expansion states (n = 289 330)
Primary cancer site		
Breast	117 751 (50.2)	155 521 (53.8)
Colorectal	50 927 (21.7)	60 793 (21.0)
Lung	65 794 (28.1)	73 016 (25.2)
Race/ethnicity		
White	169 258 (72.2)	215 058 (74.3)
Black	43 120 (18.4)	31 001 (10.7)
Hispanic	14 049 (6.0)	20 296 (7.0)
Other	8045 (3.4)	22 975 (7.9)
Female	169 130 (72.1)	216 609 (74.9)
Age, y		
40-44	19 686 (8.4)	24 956 (8.6)
45-49	32 517 (13.9)	41 742 (14.4)
50-54	50 490 (21.5)	62 874 (21.7)
55-59	62 445 (26.6)	75 271 (26.0)
60-64	69 334 (29.6)	84 487 (29.2)
Cancer stage at diagnosis		
I	80 787 (34.5)	109 598 (37.9)
II	56 555 (24.1)	69 226 (23.9)
III	42 957 (18.3)	49 844 (17.2)
IV	54 173 (23.1)	60 662 (21.0)
Insurance type		
Not insured	21 083 (9.0)	9899 (3.4)
Private	154 318 (65.8)	203 633 (70.4)
Medicaid	25 367 (10.8)	44 075 (15.2)
Medicare	27 342 (11.7)	28 327 (9.8)
Other government	6362 (2.7)	3396 (1.2)
Urban location	228 512 (97.5)	285 786 (98.8)
Charlson Comorbidity Index score		
0	178 554 (76.2)	229 702 (79.4)
1	42 135 (18.0)	45 082 (15.6)
2	9822 (4.2)	10 322 (3.6)
≥3	3961 (1.7)	4224 (1.5)
Median income by zip code, $		
<38 000	58 235 (24.8)	36 961 (12.8)
38 000-47 999	62 683 (26.7)	52 709 (18.2)
48 000-62 999	58 567 (25.0)	75 403 (26.1)
≥63 000	54 696 (23.3)	123 802 (42.8)
Missing	291 (0.1)	455 (0.2)
Less than high school education by zip code, %		
≥21.0	54 118 (23.1)	46 582 (16.1)
13.0-20.9	68 619 (29.3)	66 977 (23.1)
7.0-12.9	66 303 (28.3)	95 490 (33.0)
<7.0	45 220 (19.3)	79 946 (27.6)
Missing	212 (0.1)	335 (0.1)

Secondary models were stratified by cancer stage (stages I-III vs stage IV), cancer type, and at-risk subpopulations (ie, lowest vs highest quartiles of median household income by zip code, and Black vs White patients). We present the stratified results for these subgroups because they were a priori of clinical interest: 3-way interactions with income, race, and cancer type were not statistically significant, indicating similar changes in each set of subgroups. The 3-way interaction with stage was statistically significant. All analyses were performed from January to May 2020 using SAS statistical software version 9.4 (SAS Institute). For our primary analysis of the DID between expansion vs nonexpansion states, a 2-sided *P* < .05 was considered statistically significant. *P* values for secondary analyses should be interpreted with caution because multiple testing may produce false-positive findings. Baseline mortality trends (comparing mortality changes in preexpansion years 1 and 2 between expansion and nonexpansion states) were similar (eTable 2 in the Supplement).

For the main model, we performed sensitivity analyses examining the consistency of results separately for early and January 2014 expansion states. We ran a second set of sensitivity analyses including patients with unknown cancer stage.

## Results

### Patient Population

We identified 523 802 patients aged 40 to 64 years (385 739 women [73.6%]; mean [SD] age, 54.8 [6.5] years) with newly diagnosed invasive breast (273 272 patients [52.2%]), colorectal (111 720 patients [21.3%]), or lung (138 810 patients [26.5%]) cancer from 2012 to 2015 (eFigure 1 in the Supplement). There were 234 472 patients (44.8%) in nonexpansion states and 289 330 patients (55.2%) in Medicaid expansion states (Table 1). Most patients were White individuals and lived in an urban location. Compared with expansion states in the preexpansion period, a higher proportion of patients in nonexpansion states were uninsured (4.2% [2060 patients] in early expansion states and 5.1% [4671 patients] in 2014 expansion states vs 10.1% [11 584 patients] in nonexpansion states), were in the lowest quartile of median income by zip code (6.8% [3336 patients] in early expansion states and 15.9% [14 563 patients] in 2014 expansion states vs 25.0% [28 673 patients] in nonexpansion states), and were in the lowest quartile of education by zip code (percentage having less than a high school education, 17.6% [8634 patients] in early expansion states and 15.1% [13 831 patients] in 2014 expansion states vs 23.2% [26 609 patients] in nonexpansion states) (eTable 3 in the Supplement).

### Overall Survival

#### Total Cohort

Kaplan-Meier adjusted survival curves (Figure) and an adjusted Cox regression model (Table 2) show a small but significant mortality improvement in expansion vs nonexpansion states. The Kaplan-Meier absolute survival difference was 0.4% (meaning that 250 patients with cancer would need to gain coverage for 1 death to be prevented 4 years after cancer diagnosis). There was a statistically significant 2.0% decreased hazard of death (hazard ratio [HR], 0.98; 95% CI, 0.97-0.99; *P* = .008) in the expansion group from the preexpansion to postexpansion periods; if this 2.0% reduction in mortality were realized in all Medicaid expansion states, then among the 69 000 patients with cancer diagnosed, 1384 lives would be saved yearly. In contrast, mortality was unchanged in nonexpansion states over time (HR, 1.01; 95% CI, 0.99-1.02; *P* = .43). The DID ratio comparing the HRs in nonexpansion states with expansion states indicates that the improved mortality in expansion states was significantly different from the unchanged mortality in nonexpansion states (DID HR, 1.03; 95% CI, 1.01-1.05; *P* = .01). In general, a DID HR greater than 1 indicates a greater improvement in expansion vs nonexpansion states or less worsening in expansion vs nonexpansion states.

**Figure.**
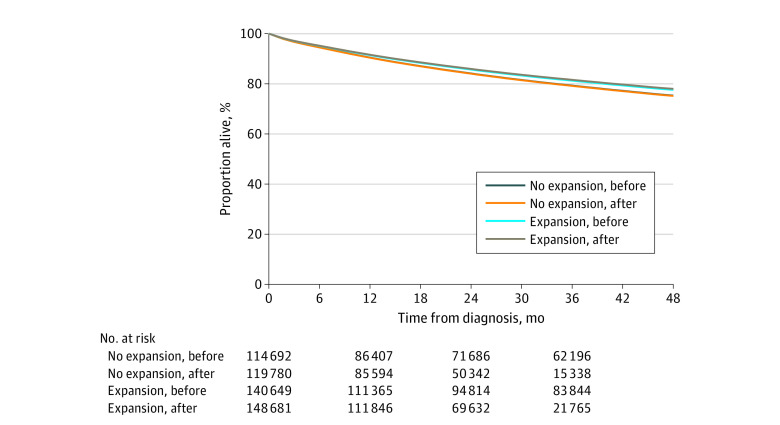
Kaplan-Meier Adjusted Survival Curves Kaplan-Meier survival curves are adjusted for age, sex, cancer type, urban location, Charlson Comorbidity Index score, and the interaction between sex and cancer type (nonexpansion states before vs after expansion, hazard ratio, 1.01; 95% CI, 0.99-1.02; *P* = .43; expansion states before vs after expansion, hazard ratio, 0.98; 95% CI, 0.97-0.99; *P* = .008).

**Table 2.** Cox Regression for Overall Cohort and Adjusted for Intermediary Variables

Model	Postexpansion vs preexpansion, HR (95% CI)	*P* value	DID HR (95% CI)^a^	*P* value
Main model				
Nonexpansion states	1.01 (0.99-1.02)	.43	1 [Reference]	NA
Combined expansion states	0.98 (0.97-0.99)	.008	1.03 (1.01-1.05)	.01
Main model adjusted for race, income, education, and insurance as intermediary variables				
Nonexpansion states	1.02 (1.01-1.04)	.003	1 [Reference]	NA
Combined expansion states	0.98 (0.96-0.99)	.006	1.05 (1.02-1.07)	<.001
Main model adjusted for stage, race, income, education, and insurance as intermediary variables				
Nonexpansion states	1.00 (0.99-1.02)	.84	1 [Reference]	NA
Combined expansion states	1.00 (0.98-1.02)	.94	1.00 (0.98-1.02)	.84

Abbreviations: DID, difference-in-difference; HR, hazard ratio; NA, not applicable.

^a^ Refers to ratio of preexpansion to postexpansion HR in nonexpansion states compared with preexpansion to postexpansion HR in combined expansion states. Ratios greater than 1 indicate more improvement in expansion states than in nonexpansion states.

Stratifying by cancer type, similar trends persisted, with expansion states having better changes over time compared with nonexpansion states (eTable 4 in the Supplement). For lung cancer, mortality improved in both expansion states (HR, 0.94; 95% CI, 0.93-0.96; *P* < .001) and nonexpansion states (HR, 0.97; 95% CI, 0.95-0.99; *P* = .003), but the improvement was significantly greater in expansion than in nonexpansion states (DID HR, 1.03; 95% CI, 1.00-1.06; *P* = .03). For breast and colorectal cancers, mortality was worse in both expansion and nonexpansion states. Although the degree to which mortality increased was lower in expansion states, the difference between the HRs was not significantly different for breast cancer (DID HR, 1.04; 95% CI, 0.98-1.10; *P* = .24) or colorectal cancer (DID HR, 1.04; 95% CI, 1.00-1.09; *P* = .08).

#### Intermediary Variables

To determine the association of intermediary variables with the decreased hazard of death, we first adjusted for education, income, insurance, and race. After adjusting for these variables, the mortality reduction persisted from the preexpansion and postexpansion periods for expansion states (HR, 0.98; 95% CI, 0.96-0.99; *P* = .006). Similarly, there was greater improvement in mortality in expansion vs nonexpansion states, which also remained significant (DID HR, 1.05; 95% CI, 1.02-1.07; *P* < .001). However, after adjusting also for cancer stage, there were no longer any reductions in mortality over time for expansion states (HR, 1.00; 95% CI, 0.98-1.02; *P* = .94). The difference between expansion and nonexpansion states in the change in the hazard of death was no longer significant after adjusting for cancer stage (DID HR, 1.00; 95% CI, 0.98-1.02; *P* = .84) (Table 2), suggesting that survival differences were mediated by cancer stage.

#### Cancer Stage

For patients with stages I to III (nonmetastatic) cancer, there was a significant 4.8% increased hazard of death in nonexpansion states (HR, 1.05; 95% CI, 1.02-1.08; *P* < .001) and unchanged mortality in expansion states from the preexpansion to postexpansion period (HR, 0.99; 95% CI, 0.97-1.02; *P* = .64) (Table 3). There was significantly improved mortality in expansion vs nonexpansion states (DID HR, 1.05; 95% CI, 1.02-1.09; *P* = .003). For patients with stage IV (metastatic) cancer, both expansion and nonexpansion states had mortality improvements, but the differences were not significant. The association of Medicaid expansion was significantly different among patients with stages I to III cancer compared with patients with stage IV cancer (3-way DID HR, 1.05 [95% CI, 1.02-1.09] for stages I-III vs 0.98 [95% CI, 0.95-1.00] for stage IV; *P* = .002).

**Table 3.** Cox Regression by Cancer Stage at Diagnosis

Cancer stage	Postexpansion vs preexpansion, HR (95% CI)	*P* value	DID HR (95% CI)^a^	*P* value
I-III (nonmetastatic)				
Nonexpansion states	1.05 (1.02-1.08)	<.001	1 [Reference]	NA
Combined expansion states	0.99 (0.97-1.02)	.64	1.05 (1.02-1.09)^b^	.003
IV (metastatic)				
Nonexpansion states	0.97 (0.95-0.99)	.003	1 [Reference]	NA
Combined expansion states	0.99 (0.98-1.01)	.52	0.98 (0.95-1.00)^b^	.08

Abbreviations: DID, difference-in-difference; HR, hazard ratio; NA, not applicable.

^a^ Refers to ratio of preexpansion to postexpansion HR in nonexpansion states compared with preexpansion to postexpansion HR in combined expansion states. Ratios greater than 1 indicate more improvement in expansion states than in nonexpansion states.

^b^ A 3-way interaction term indicates that the mortality benefit among patients with stages I to III in combined expansion states compared with nonexpansion states is significantly different from the mortality among patients with stage IV cancer in nonexpansion states compared with combined expansion states (DID HR, 1.05 [95% CI, 1.02-1.09] vs 0.98 [95% CI, 0.95-1.00]; *P* = .002).

#### At-Risk Populations

Among patients living in areas with the lowest quartile of median household income, mortality was unchanged within nonexpansion states (HR, 1.00; 95% CI, 0.97-1.03; *P* = .91) and decreased only slightly and not significantly for expansion states over time (HR, 0.99; 95% CI, 0.95-1.02; *P* = .43) (Table 4 and eFigure 2 in the Supplement). These mortality changes were not significantly different (DID HR, 1.02; 95% CI, 0.97-1.06; *P* = .48). Among patients living in areas with the highest quartile of median household income, mortality increased slightly but not significantly within nonexpansion states (HR, 1.03; 95% CI, 0.99-1.07; *P* = .19) and decreased slightly but not significantly within expansion states (HR, 0.98; 95% CI, 0.96-1.01; *P* = .23); again, the mortality changes were not significantly different (DID HR, 1.04; 95% CI, 1.00-1.09; *P* = .07). Medicaid expansion was not associated with a difference in mortality among patients in the lowest vs highest quartile of median household income (DID HR, 1.02 [95% CI, 0.97-1.06] for the lowest quartile vs 1.04 [95% CI, 1.00-1.09] for the highest quartile; *P* = .49).

**Table 4.** Cox Regression Models by At-Risk Populations

Variable	Postexpansion vs preexpansion, HR (95% CI)	*P* value	DID HR (95% CI)^a^	*P* value
Lowest quartile of income by zip code				
Nonexpansion states	1.00 (0.97-1.03)	.91	1 [Reference]	NA
Combined expansion	0.99 (0.95-1.02)	.43	1.02 (0.97-1.06)^b^	.48
Highest quartile of income by zip code				
Nonexpansion states	1.03 (0.99-1.07)	.19	1 [Reference]	NA
Combined expansion states	0.98 (0.96-1.01)	.23	1.04 (1.00-1.09)^b^	.73
Black patients				
Nonexpansion states	1.00 (0.97-1.04)	.95	1 [Reference]	NA
Combined expansion states	0.97 (0.93-1.01)	.10	1.04 (0.98-1.09)^c^	.19
White patients				
Nonexpansion states	1.02 (1.00-1.04)	.09	1 [Reference]	NA
Combined expansion states	0.99 (0.97-1.01)	.17	1.03 (1.00-1.05)^c^	.03

Abbreviations: DID, difference-in-difference; HR, hazard ratio; NA, not applicable.

^a^ Refers to ratio of preexpansion to postexpansion HR in nonexpansion states compared with preexpansion to postexpansion HR in combined expansion states. Ratios greater than 1 indicate more improvement in expansion states than in nonexpansion states.

^b^ A 3-way interaction term indicates that the mortality benefit among residents in low-income neighborhoods in combined expansion states compared with nonexpansion states is not significantly different from the benefit among residents in high income neighborhoods in combined expansion states compared with nonexpansion states (DID HR, 1.02 [95% CI, 0.97-1.06] vs 1.04 [95% CI, 1.00-1.09]; *P* = .49).

^c^ A 3-way interaction term indicates that the mortality benefit among Black residents in combined expansion states compared with nonexpansion states is not significantly different from the benefit among White residents in combined expansion states compared with nonexpansion states (DID HR, 1.04 [95% CI, 0.98-1.09] vs 1.03 [95% CI, 1.00-1.05]; *P* = .74).

For Black patients, mortality decreased slightly but not significantly in expansion states (HR, 0.97; 95% CI, 0.93-1.01; *P* = .10) and was unchanged in nonexpansion states (HR, 1.00; 95% CI, 0.97-1.04; *P* = .95) from the preexpansion to postexpansion period. These mortality change differences were not significant (DID HR, 1.04; 95% CI, 0.98-1.09; *P* = .19), although the small samples sizes limited statistical power. For White patients, mortality was slightly but not significantly lower in expansion states (HR, 0.99; 95% CI, 0.97-1.01; *P* = .17) and slightly but not significantly higher in nonexpansion states (HR, 1.02; 95% CI, 1.00-1.04; *P* = .09). However, these mortality changes were significantly different in the DID analysis (DID HR, 1.03; 95% CI, 1.00-1.05; *P* = .03). Medicaid expansion was not associated with a difference in mortality between Black vs White patients (DID HR, 1.04 [95% CI, 0.98-1.09] for Black patients vs 1.03 [95% CI, 1.00-1.05] for White patients; *P* = .74).

### Sensitivity Analyses

With our first sensitivity analysis, we stratified expansion states into early and January 2014 groups (eTable 5 in the Supplement). The DID ratio comparing HRs in nonexpansion vs early expansion states indicated that the improved mortality in the early expansion states was significantly different from the unchanged mortality in nonexpansion states (DID HR, 1.04; 95% CI, 1.01-1.07; *P* = .01). Similarly, there was improved mortality in the January 2014 expansion states compared with nonexpansion states, although the difference was not significant (DID HR, 1.02; 95% CI, 1.00-1.05; *P* = .08). We ran a second set of sensitivity analyses including patients with unknown stage, and the findings were consistent with our primary findings (eTable 6 in the Supplement).

## Discussion

In this national study of patients with invasive breast, colorectal, and lung cancer, Medicaid expansion was associated with a significant decrease in mortality in expansion states compared with nonexpansion states. The decreased mortality may be due to increases in early-stage diagnosis associated with better access to screening and early diagnosis of cancer. There were no differences in mortality changes among at-risk populations, including among Black patients and among those living in areas with the lowest quartile of income.

There are several factors that could explain improved mortality associated with Medicaid expansion. Prior research^9^ demonstrated a small but significant increase in early-stage diagnoses in expansion states compared with nonexpansion states. Our analyses that omitted and then included cancer stage suggest a plausible association wherein earlier stage at diagnosis appears to explain the mortality improvement. Increased Medicaid coverage may remove barriers to accessing the health care system for screening and timely symptom evaluation, especially because we typically consider insurance coverage as an immediate change once implemented. The lower mortality in expansion states compared with nonexpansion states was similar across all 3 cancer types, although in stratified analyses, the difference was significant only for lung cancer. Lung cancer has a higher mortality rate than breast and colorectal cancer, and with longer follow-up, it is possible that the lower mortality rates seen for breast and colorectal cancer may also become significant. In addition, in the US, it appears that there has been a dramatic improvement in lung cancer mortality during this period, whereas the improvements in breast and colorectal cancer mortality have slowed over the years.^21^

Prior studies^22,23^ of Medicaid expansion in 2000 and Massachusetts health reform in 2006 demonstrated lower death rates after expansion. In the 2000 Medicaid expansion study,^22^ the authors estimated that 1 death was prevented for every 239 to 316 adults who gained coverage, whereas the Massachusetts 2006 health reform study^23^ found 1 death per year prevented for 830 adults who gained coverage. In the present study, on the basis of the Kaplan-Meier absolute survival difference of 0.4%, we estimate that 250 patients with cancer would need to gain coverage for 1 death to be prevented 4 years after cancer diagnosis. Moreover, if the 2.0% absolute reduction in mortality were realized in all Medicaid expansion states (early and January 2014 expansion states), then among the approximately 69 000 patients with cancer diagnosed, 1384 lives would be saved yearly. Although the comparisons are not straightforward given the different populations and periods, this provides a concrete sense of lives saved by Medicaid coverage for patients with cancer.

When we examined the mortality among patients with curable (stages I-III) and metastatic (stage IV) cancer, we saw a significant mortality improvement in expansion states for patients with curable cancer but not those with metastatic cancer after Medicaid expansion. Interestingly, mortality in the metastatic cohort improved similarly for both expansion and nonexpansion states. It is possible that increased development and use of immunotherapies has decreased mortality in most of these groups over time, especially in the metastatic setting, for which most immunotherapies are initially tested and approved.^24^ Also, with increased focus and attention on palliative care, it is possible that patients with metastatic disease are receiving better end-of-life care^25^ in both expansion and nonexpansion states.

Although the main goal of our study was to understand the mortality difference between expansion and nonexpansion states after expansion, we saw a baseline mortality difference between the 2 groups, with patients with cancer in nonexpansion states having a higher hazard of death; this preexisting mortality gap persisted in the postexpansion period. Potential hypotheses include differences in social support, social determinants of health, or limited health resources. Both expansion and nonexpansion states saw an increase in the total number of patients with breast, colorectal, or lung cancer from the preexpansion to postexpansion period. However, it is possible that expansion states were better prepared for the increase in patients given advanced notice of expanding Medicaid and anticipating a potential increase in patients using hospital resources. Additional studies are warranted to further understand these baseline differences.

Any new policy may result in unintended consequences, especially for at-risk populations. Prior studies have highlighted disparities in outcomes for patients with cancer.^26,27,28^ We were reassured to find that patients in areas in the lowest quartile of median household income within expansion states showed a modest, nonsignificant decrease in mortality after expansion. We also observed that mortality improvements associated with Medicaid expansion did not differ for Black vs White patients.

Our work adds to a limited body of evidence on Medicaid expansion and cancer care. A few studies have shown that among patients with cancer, expansion was associated with increased insurance coverage,^6,9,29^ earlier stage of diagnosis,^9,30,31^ and no changes in timeliness of treatment.^9,32^ Although some studies^33,34^ have investigated the association of Medicaid expansion with mortality for some disease processes (ie, end-stage renal disease or cardiovascular disease), to our knowledge, there are no studies that investigate the association between Medicaid expansion and cancer mortality.

### Limitations

There are several limitations of our study. First, this is an observational study. Randomized clinical trials of new policies are often difficult to implement. We attempted to minimize unmeasured confounding by using a DID analysis. Second, we lacked patient-level information regarding several variables, including individuals’ state of residence or eligibility for Medicaid, meaning that we were unable to control for state-level fixed effects, income, and education. Third, our study focused on patients with breast, colorectal, and lung cancer aged 40 to 64 years. This could limit the generalizability of our data, although few patients with cancers of these types receive a diagnosis before age 40 years. Fourth, we examined time to death, which is subject to lead-time bias if increases in screening preferentially detect slower-growing tumors. However, when analyzing mortality by cancer type, we found that although all groups had lower mortality within expansion vs nonexpansion states, those with breast and colorectal cancer saw a slight decrease in time to death over time (in both expansion and nonexpansion groups), which would not be consistent with our findings being explained by increases in cancer screening. Furthermore, longer follow-up would be helpful to understand whether this mortality improvement persists.

## Conclusions

This cross-sectional study found that Medicaid expansion was associated with improved mortality in patients with newly diagnosed breast, lung, and colorectal cancers. This association appeared to be mediated by earlier stage of cancer diagnosis and did not differ by race or area-level income.

## Notes

Supplement. **eAppendix 1.** Definition of First Cancer Diagnosis **eTable 1.** National Cancer Database Medicaid Expansion Status Variable **eAppendix 2.** Definition of Race Variable **eFigure 1.** Population Flowchart **eTable 2.** Baseline Trend for DID Assumption **eTable 3.** Baseline Characteristics for Pre- and Post-Expansion Groups **eTable 4.** Cox Regression by Cancer Type **eFigure 2.** Forest Plot of Hazard Ratios by Vulnerable Population **eTable 5.** Cox Regression by Early and January 2014 Expansion Groups **eTable 6.** Cox Regression of Patients with Missing/Unknown Stage

## References

[1] Kaiser Family Foundation Current status of states Medicaid expansion decisions. Kaiser Health News. Published 2020 Accessed March 18, 2020. https://www.kff.org/medicaid/issue-brief/status-of-state-medicaid-expansion-decisions-interactive-map/

[2] Frean M, Gruber J, Sommers BD Disentangling the ACA’s coverage effects: lessons for policymakers. N Engl J Med. 2016;375(17):1605-1608. doi:10.1056/NEJMp1609016 27653467

[3] Choi SK, Adams SA, Eberth JM, Medicaid coverage expansion and implications for cancer disparities. Am J Public Health. 2015;105(5)(suppl):S706-S712. doi:10.2105/AJPH.2015.302876 26447909 PMC4627517

[4] Jemal A, Lin CC, Davidoff AJ, Han X Changes in insurance coverage and stage at diagnosis among nonelderly patients with cancer after the Affordable Care Act. J Clin Oncol. 2017;35(35):3906-3915. doi:10.1200/JCO.2017.73.7817 28885865

[5] Han X, Yabroff KR, Ward E, Brawley OW, Jemal A Comparison of insurance status and diagnosis stage among patients with newly diagnosed cancer before vs after implementation of the Patient Protection and Affordable Care Act. JAMA Oncol. 2018;4(12):1713-1720. doi:10.1001/jamaoncol.2018.3467 30422152 PMC6440711

[6] Soni A, Sabik LM, Simon K, Sommers BD Changes in insurance coverage among cancer patients under the Affordable Care Act. JAMA Oncol. 2018;4(1):122-124. doi:10.1001/jamaoncol.2017.3176 29049486 PMC5833642

[7] Fedewa SA, Yabroff KR, Smith RA, Goding Sauer A, Han X, Jemal A Changes in breast and colorectal cancer screening after Medicaid expansion under the Affordable Care Act. Am J Prev Med. 2019;57(1):3-12. doi:10.1016/j.amepre.2019.02.015 31128952

[8] Gan T, Sinner HF, Walling SC, Impact of the Affordable Care Act on colorectal cancer screening, incidence, and survival in Kentucky. J Am Coll Surg. 2019;228(4):342-353.e1. doi:10.1016/j.jamcollsurg.2018.12.035 30802505 PMC6537585

[9] Takvorian SU, Oganisian A, Mamtani R, Association of Medicaid expansion under the Affordable Care Act with insurance status, cancer stage, and timely treatment among patients with breast, colon, and lung cancer. JAMA Netw Open. 2020;3(2):e1921653. doi:10.1001/jamanetworkopen.2019.21653 32074294 PMC12549135

[10] Sommers BD, Gawande AA, Baicker K Health insurance coverage and health: what the recent evidence tells us. N Engl J Med. 2017;377(6):586-593. doi:10.1056/NEJMsb1706645 28636831

[11] Sommers BD, Gunja MZ, Finegold K, Musco T Changes in self-reported insurance coverage, access to care, and health under the Affordable Care Act. JAMA. 2015;314(4):366-374. doi:10.1001/jama.2015.8421 26219054

[12] Crocker AB, Zeymo A, McDermott J, Expansion coverage and preferential utilization of cancer surgery among racial and ethnic minorities and low-income groups. Surgery. 2019;166(3):386-391. doi:10.1016/j.surg.2019.04.018 31213307

[13] Zerhouni YA, Trinh QD, Lipsitz S, Effect of Medicaid expansion on colorectal cancer screening rates. Dis Colon Rectum. 2019;62(1):97-103. doi:10.1097/DCR.0000000000001260 30407931

[14] Al-Refaie WB, Zheng C, Jindal M, Did pre-Affordable Care Act Medicaid expansion increase access to surgical cancer care? J Am Coll Surg. 2017;224(4):662-669. doi:10.1016/j.jamcollsurg.2016.12.044 28130171 PMC5698907

[15] Xiao D, Zheng C, Jindal M, Medicaid expansion and disparity reduction in surgical cancer care at high-quality hospitals. J Am Coll Surg. 2018;226(1):22-29. doi:10.1016/j.jamcollsurg.2017.09.012 28987635 PMC5742300

[16] Yang W, Williams JH, Hogan PF, Projected supply of and demand for oncologists and radiation oncologists through 2025: an aging, better-insured population will result in shortage. J Oncol Pract. 2014;10(1):39-45. doi:10.1200/JOP.2013.001319 24443733

[17] Nass S, Patlak M, Zevon E, Balought E Developing and sustaining an effective and resilient oncology careforce: a workshop. National Academy of Medicine. Published 2019 Accessed October 2, 2020. https://www.nationalacademies.org/our-work/developing-and-sustaining-an-effective-and-resilient-oncology-careforce-a-workshop

[18] Miller S, Wherry LR Health and access to care during the first 2 years of the ACA Medicaid expansions. N Engl J Med. 2017;376(10):947-956. doi:10.1056/NEJMsa1612890 28273021

[19] Mazurenko O, Balio CP, Agarwal R, Carroll AE, Menachemi N The effects of Medicaid expansion under the ACA: a systematic review. Health Aff (Millwood). 2018;37(6):944-950. doi:10.1377/hlthaff.2017.1491 29863941

[20] von Elm E, Altman DG, Egger M, Pocock SJ, Gøtzsche PC, Vandenbroucke JP; STROBE Initiative The Strengthening the Reporting of Observational Studies in Epidemiology (STROBE) statement: guidelines for reporting observational studies. Lancet. 2007;370(9596):1453-1457. doi:10.1016/S0140-6736(07)61602-X 18064739

[21] Siegel RL, Miller KD, Jemal A Cancer statistics, 2020. CA Cancer J Clin. 2020;70(1):7-30. doi:10.3322/caac.21590 31912902

[22] Sommers BD, Baicker K, Epstein AM Mortality and access to care among adults after state Medicaid expansions. N Engl J Med. 2012;367(11):1025-1034. doi:10.1056/NEJMsa1202099 22830435

[23] Sommers BD, Long SK, Baicker K Changes in mortality after Massachusetts health care reform: a quasi-experimental study. Ann Intern Med. 2014;160(9):585-593. doi:10.7326/M13-2275 24798521

[24] Sharma P, Wagner K, Wolchok JD, Allison JP Novel cancer immunotherapy agents with survival benefit: recent successes and next steps. Nat Rev Cancer. 2011;11(11):805-812. doi:10.1038/nrc3153 22020206 PMC3426440

[25] Temel JS, Greer JA, Muzikansky A, Early palliative care for patients with metastatic non-small-cell lung cancer. N Engl J Med. 2010;363(8):733-742. doi:10.1056/NEJMoa1000678 20818875

[26] O’Keefe EB, Meltzer JP, Bethea TN Health disparities and cancer: racial disparities in cancer mortality in the United States, 2000-2010. Front Public Health. 2015;3:51. doi:10.3389/fpubh.2015.00051 25932459 PMC4398881

[27] Lucas FL, Stukel TA, Morris AM, Siewers AE, Birkmeyer JD Race and surgical mortality in the United States. Ann Surg. 2006;243(2):281-286. doi:10.1097/01.sla.0000197560.92456.32 16432363 PMC1448914

[28] Wheeler SB, Reeder-Hayes KE, Carey LA Disparities in breast cancer treatment and outcomes: biological, social, and health system determinants and opportunities for research. Oncologist. 2013;18(9):986-993. doi:10.1634/theoncologist.2013-0243 23939284 PMC3780646

[29] Moss HA, Havrilesky LJ, Zafar SY, Suneja G, Chino J Trends in insurance status among patients diagnosed with cancer before and after implementation of the Affordable Care Act. J Oncol Pract. 2018;14(2):e92-e102. doi:10.1200/JOP.2017.027102 29436303

[30] Soni A, Simon K, Cawley J, Sabik L Effect of Medicaid expansions of 2014 on overall and early-stage cancer diagnoses. Am J Public Health. 2018;108(2):216-218. doi:10.2105/AJPH.2017.304166 29267058 PMC5846584

[31] Davidoff AJ, Guy GP Jr, Hu X, Changes in health insurance coverage associated with the Affordable Care Act among adults with and without a cancer history: population-based national estimates. Med Care. 2018;56(3):220-227. doi:10.1097/MLR.0000000000000876 29438192 PMC6105312

[32] Adamson BJS, Cohen AB, Estevez M, Affordable Care Act (ACA) Medicaid expansion impact on racial disparities in time to cancer treatment. J Clin Oncol. 2019;37(18)(suppl):LBA1. doi:10.1200/jco.2019.37.18_suppl.lba1

[33] Wadhera RK, Bhatt DL, Wang TY, Association of state Medicaid expansion with quality of care and outcomes for low-income patients hospitalized with acute myocardial infarction. JAMA Cardiol. 2019;4(2):120-127. doi:10.1001/jamacardio.2018.4577 30649146 PMC6439625

[34] Swaminathan S, Sommers BD, Thorsness R, Mehrotra R, Lee Y, Trivedi AN Association of Medicaid expansion with 1-year mortality among patients with end-stage renal disease. JAMA. 2018;320(21):2242-2250. doi:10.1001/jama.2018.16504 30422251 PMC6417808

