# Supplementary material for: Medicaid Expansion and Mortality Among Patients With Breast, Lung, and Colorectal Cancer
Source: JAMA Netw Open. 2020 Nov 5;3(11):e2024366. doi: 10.1001/jamanetworkopen.2020.24366 (PMC7645694; doi:10.1001/jamanetworkopen.2020.24366)
Supplement: Supplement. — eAppendix 1. Definition of First Cancer Diagnosis eTable 1. National Cancer Database Medicaid Expansion Status Variable eAppendix 2. Definition of Race Variable eFigure 1. Population Flowchart eTable 2. Baseline Trend for DID Assumption eTable 3. Baseline Characteristics for Pre- and Post-Expansion Groups eTable 4. Cox Regression by Cancer Type eFigure 2. Forest Plot of Hazard Ratios by Vulnerable Population eTable 5. Cox Regression by Early and January 2014 Expansion Groups eTable 6. Cox Regression of Patients with Missing/Unknown Stage [file jamanetwopen-e2024366-s001.pdf]

Supplemental Online Content

Lam MB, Phelan J, Orav EJ, Jha AK, Keating NL. Medicaid expansion and mortality among patients with breast, lung, and colorectal cancer. *JAMA Netw Open*. 2020;3(11):e2024366. doi:10.1001/jamanetworkopen.2020.24366

eAppendix 1. Definition of First Cancer Diagnosis
eTable 1. National Cancer Database Medicaid Expansion Status Variable
eAppendix 2. Definition of Race Variable
eFigure 1. Population Flowchart
eTable 2. Baseline Trend for DID Assumption
eTable 3. Baseline Characteristics for Pre- and Post-Expansion Groups
eTable 4. Cox Regression by Cancer Type
eFigure 2. Forest Plot of Hazard Ratios by Vulnerable Population
eTable 5. Cox Regression by Early and January 2014 Expansion Groups
eTable 6. Cox Regression of Patients with Missing/Unknown Stage

This supplemental material has been provided by the authors to give readers additional information about their work.

© 2020 Lam MB et al. *JAMA Network Open.*

eAppendix 1. Definition of First Cancer Diagnosis

National Cancer Database variable "Sequence Number" defines whether a new cancer diagnosis is their only lifetime diagnosis (00) and/or their first diagnosis (01). A prior study included 00 and 01 for their cohort and therefore we also follow a similar methodology. For the majority of patients (>95%), this was their only lifetime cancer diagnosis and for the remaining patients (<5%) this was their first cancer diagnosis. The results did not change when we excluded the <5% group.

"The data item Sequence Number refers to the sequence of malignant and non-malignant tumors diagnosed in a patient and is used to distinguish cases with multiple cancer diagnoses. By default, your PUF includes all sequence codes available for each reported patient. Patients with only one lifetime cancer diagnosis will have a sequence number code value of 00. Sequence number 01 indicates that the reported tumor is the first of multiple diagnoses. The NCDB has no mechanism by which to link separate case reports of the same patient. It is customary to limit analyses to patients with sequence numbers 00 and 01 to ensure that any review of treatment or outcomes of the study cohort is not confounded by treatment administered for a prior cancer diagnosis…"

Excluding the <5%

	**Post- to Pre-Expansion Hazard Ratio (95% Confidence Interval) and p-value**	**Difference-in-Difference Ratio* and p-value**
**Main Model – Excluding <5% of Patients with Sequence 01**		
Non-Expansion	0.99 (0.97-1.01)<br>p=0.22	Reference
Combined Expansion	0.97 (0.95-0.98)<br>p<0.001	1.03 (1.00-1.05)<br>p=0.03

© 2020 Lam MB et al. *JAMA Network Open.*

eTable 1. National Cancer Database Medicaid Expansion Status Variable

Definition	States Included
Non-Expansion (Control) States	TN, NC, ID, GA, FL, MO, AL, MS, KS, TX, WI, UT, SC, SD, VA, OK, NE, WY, ME
January 2014 Expansion States	KY, NV, CO, OR, NM, WV, AR, RI, AZ, MD, MA, ND, OH, IA, IL, VT, HI, NY, DE
Early Expansion States (2010-2013)	WA, CA, NJ, MN, DC, CT
Late Expansion States (after Jan. 2014)*	NH, IN, MI, PA, AK, MT, LA

*Late expansion states were excluded from our study to allow to enough follow up time to assess mortality

© 2020 Lam MB et al. *JAMA Network Open.*

eAppendix 2. Definition of Race Variable

Race categories were defined using a combination the NCDB race and Hispanic origin variables. If any Spanish/Hispanic origin is indicated, regardless of race, they are coded as “Hispanic”. If they are White (from race) and non-Hispanic/non-Spanish (from Hispanic origin) they are coded as non-Hispanic “White”. If they are Black (from race) and non-Hispanic/non-Spanish (from Hispanic origin) they are coded as Non-Hispanic “Black”. All others not falling into one of those three categories are coded as “Other”.

© 2020 Lam MB et al. *JAMA Network Open.*

eFigure 1. Population Flowchart

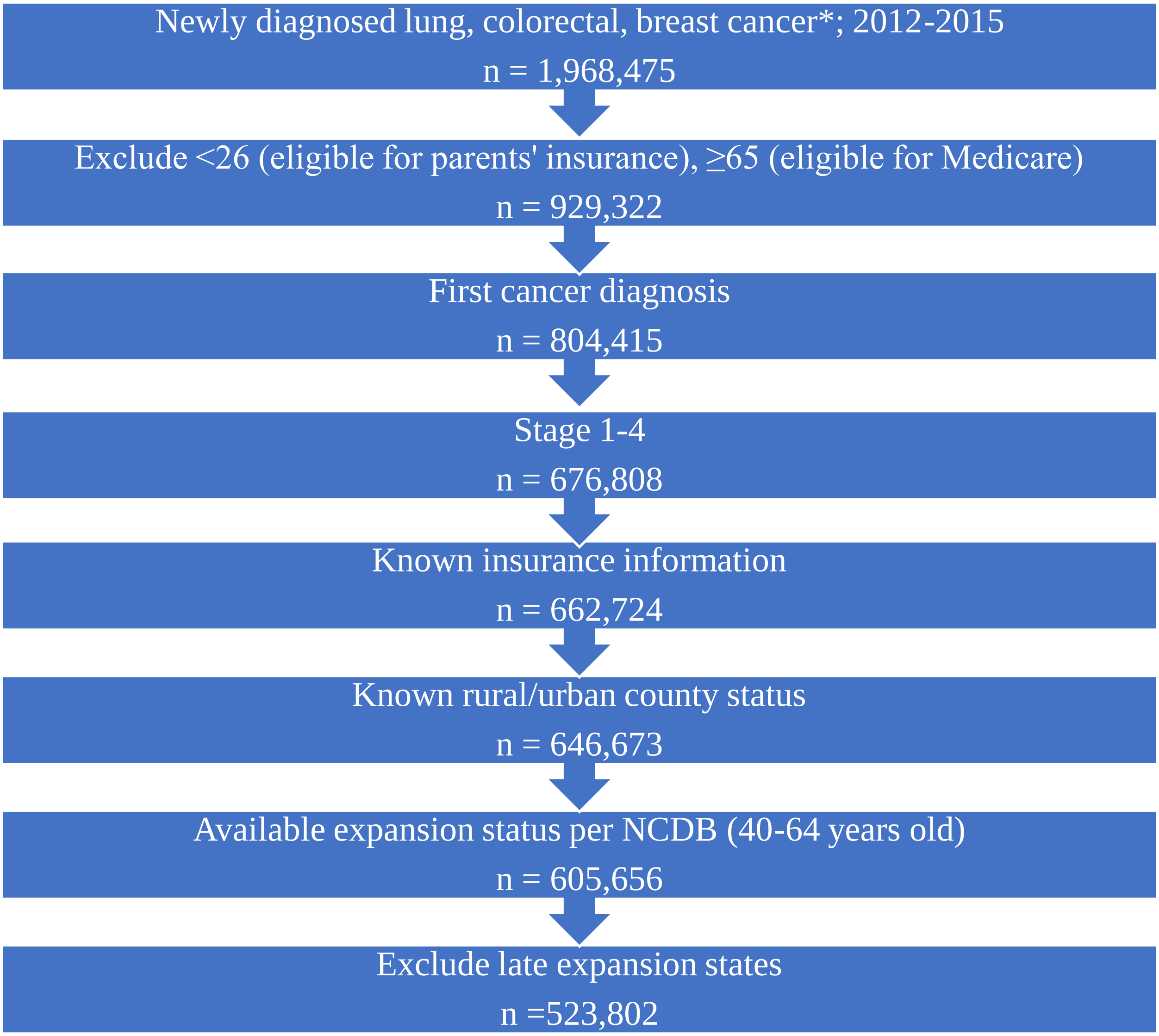

* International Classification of Disease for Oncology, Third Edition codes were used to select patients: C500-509 (breast), C180-189, C260, C199, C209 (colorectal), C340-349 (lung).

© 2020 Lam MB et al. *JAMA Network Open.*

eTable 2. Baseline Trend for DID Assumption*

	**Pre-Year 2 to Pre-Year 1 Hazard Ratio (95% Confidence Interval) and p-value**	**Difference-in-Difference Ratio** and p-value**
Non-Expansion	1.002 (0.982-1.023)<br>p<0.001	Reference
Combined Expansion	1.024 (1.004-1.045)<br>p=0.018	0.979 (0.951-1.007)<br>p=0.134

*A key assumption for DID models is that baseline trends between intervention and comparison groups are similar.
**Ratio of pre-year 1 to pre-year 2 HR in non-expansion states compared to pre-year 1 to pre-year 2 HR in combined expansion states. Ratios greater than 1 indicate more improvement in expansion states than in non-expansion states.

© 2020 Lam MB et al. *JAMA Network Open.*

eTable 3. Baseline Characteristics for Pre- and Post-Expansion Groups

	Pre-Expansion (Jan 2012-Dec 2013)			Post-Expansion (Jan 2014-Dec 2016)		
	**Non-Expansion**	**Early Expansion**	**Jan 2014 Expansion**	**Non-Expansion**	**Early Expansion**	**Jan 2014 Expansion**
**N**	114,692	49,055	91,594	119,780	51,959	96,722
**Primary Cancer Site**						
Breast	50.0%	57.7%	51.8%	50.4%	57.2%	51.7%
Colorectal	21.4%	21.2%	20.5%	22.0%	21.9%	20.9%
Lung	28.6%	21.1%	27.7%	27.6%	20.9%	27.4%
**Race/Ethnicity**						
White	72.5%	70.0%	77.4%	71.9%	68.7%	76.6%
Black	18.3%	8.9%	11.7%	18.5%	8.8%	11.7%
Hispanic	5.9%	10.7%	4.7%	6.0%	11.2%	5.1%
Other	3.3%	10.5%	6.2%	3.6%	11.3%	6.5%
**Female**	72.0%	77.1%	73.8%	72.3%	76.8%	73.7%
**Age**						
40-44	8.5%	9.3%	8.5%	8.3%	9.1%	8.2%
45-49	14.3%	15.6%	14.4%	13.4%	14.6%	13.7%
50-54	21.9%	22.0%	22.1%	21.2%	21.8%	21.3%
55-59	26.1%	25.3%	25.8%	27.1%	25.8%	26.6%
60-64	29.2%	27.8%	29.2%	29.9%	28.7%	30.2%
**Stage at Diagnosis**						
I	34.0%	38.0%	36.9%	34.9%	39.0%	38.1%
II	24.2%	25.3%	23.3%	24.0%	25.1%	23.1%
III	18.6%	17.1%	17.6%	18.1%	16.7%	17.1%
IV	23.3%	19.5%	22.1%	23.0%	19.2%	21.6%
**Insurance Type**						
Not Insured	10.1%	4.2%	5.1%	7.9%	2.1%	2.2%
Private	64.3%	72.1%	69.8%	67.2%	72.5%	69.0%
Medicaid	11.5%	13.4%	13.6%	10.2%	16.1%	17.2%
Medicare	11.4%	9.3%	10.3%	11.9%	8.4%	10.3%
Other Gov't	2.7%	1.0%	1.2%	2.7%	0.9%	1.3%
**Urban Location**	97.4%	99.5%	98.4%	97.5%	99.5%	98.4%
**Charlson Comorbidity Score**						
0	75.8%	83.0%	77.9%	76.5%	81.4%	77.9%
1	18.4%	13.3%	16.6%	17.6%	14.5%	16.4%
2	4.2%	2.6%	3.9%	4.2%	2.9%	4.0%

© 2020 Lam MB et al. *JAMA Network Open.*

3+	1.6%	1.1%	1.6%	1.7%	1.3%	1.7%
**Median Income by Zipcode (Quartiles)**						
< $38,000	25.0%	6.8%	15.9%	24.7%	6.8%	16.1%
$38,000-$47,999	26.8%	12.7%	20.9%	26.7%	13.2%	21.2%
$48,000-$62,999	24.8%	26.3%	25.7%	25.1%	26.4%	26.0%
>=$63,000	23.2%	54.0%	37.3%	23.4%	53.5%	36.5%
Missing	0.1%	0.1%	0.2%	0.1%	0.1%	0.2%
**Education: Less than High School by Zipcode (Quartiles)**						
>=21.0%	23.2%	17.6%	15.1%	23.0%	17.4%	15.6%
13.0-20.9%	29.3%	18.3%	25.4%	29.2%	18.9%	25.7%
7.0-12.9%	28.3%	33.2%	33.0%	28.3%	33.2%	32.8%
<7.0%	19.1%	30.8%	26.4%	19.4%	30.4%	25.7%
Missing	0.1%	0.1%	0.1%	0.1%	0.1%	0.1%

© 2020 Lam MB et al. *JAMA Network Open.*

eTable 4. Cox Regression by Cancer Type

	Post- to Pre-Expansion Hazard Ratio (95% Confidence Interval) and p-value	Difference-in-Difference Ratio* and p-value
**Breast**		
Non-Expansion	1.117 (1.069-1.167)<br>p<0.001	Reference
Combined Expansion	1.078 (1.034-1.125)<br>p<0.001	1.036** (0.977-1.098)<br>p=0.240
**Colorectal**		
Non-Expansion	1.096 (1.058-1.135)<br>p<0.001	Reference
Combined Expansion	1.051 (1.016-1.087)<br>p=0.004	1.043** (0.995-1.093)<br>p=0.083
**Lung**		
Non-Expansion	0.972 (0.953-0.990)<br>p=0.003	Reference
Combined Expansion	0.944 (0.927-0.962)<br>p<0.001	1.029** (1.002-1.057)<br>p=0.033

*Ratio of pre- to post- HR in non-expansion states compared to pre to post HR in combined expansion states. Ratios greater than 1 indicate more improvement in expansion states than in non-expansion states.

** A three-way interaction term indicates that the mortality benefit among breast cancer patients in combined expansion states relative to non-expansion states (DID-HR 1.036) is not significantly different (p=0.911) from the benefit among colorectal cancer patients in combined expansion states relative to non-expansion states (DID-HR 1.043) or from lung cancer patients in combined expansion states relative to non-expansion states (DID-HR 1.029).

© 2020 Lam MB et al. *JAMA Network Open.*

eFigure 2. Forest Plot of Hazard Ratios by Vulnerable Population

Hazard Ratios Post Medicaid Expansion (Relative to Pre Period)

Patient Group	HR (95% CI)	P value
**Low Income Counties**		
Non-expansion States	1.0017 (0.9741-1.0301)	0.905
Expansion States	0.9860 (0.9519-1.0213)	0.432
**High Income Counties**		
Non-expansion States	1.0271 (0.9872-1.0686)	0.186
Expansion States	0.9838 (0.9576-1.0106)	0.234
**Black**		
Non-expansion States	0.9999 (0.9665-1.0345)	0.996
Expansion States	0.9648 (0.9250-1.0063)	0.096
**White**		
Non-expansion States	1.0162 (0.9978-1.0349)	0.085
Expansion States	0.9881 (0.9712-1.0053)	0.173

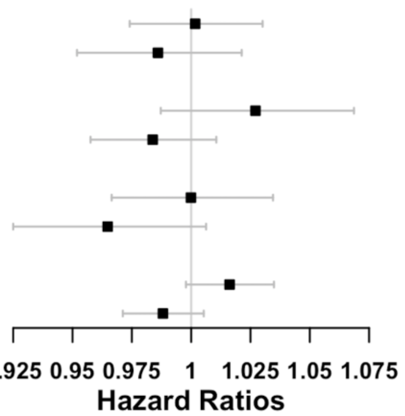

© 2020 Lam MB et al. *JAMA Network Open.*

eTable 5. Cox Regression by Early and January 2014 Expansion Groups

	Post- to Pre-Expansion Hazard Ratio (95% Confidence Interval) and p-value	Difference-in-Difference Ratio* and p-value
**Main Model**		
Non-Expansion	1.006 (0.991-1.022) p=0.431	Reference
Combined Expansion	0.980 (0.965-0.995) p=0.008	1.027 (1.005-1.049) p=0.015
Early Expansion	0.968 (0.942-0.994) p=0.016	1.040 (1.008-1.072) p=0.013
Jan 2014 Expansion	0.985 (0.968-1.003) p=0.110	1.021 (0.997-1.046) p=0.080

© 2020 Lam MB et al. *JAMA Network Open.*

eTable 6. Cox Regression of Patients with Missing/Unknown Stage

	**Post- to Pre-Expansion Hazard Ratio (95% Confidence Interval) and p-value**	**Difference-in-Difference Ratio* and p-value**
**Main Model – Including Missing/Unknown Stage**		
Non-Expansion	1.005 (0.989-1.020)<br>p=0.541	Reference
Combined Expansion	0.977 (0.963-0.992)<br>p=0.003	1.028 (1.007-1.050)<br>p=0.010
**Main Model – Missing/Unknown Stage Only**		
Non-Expansion	0.951 (0.856-1.057)<br>p=0.355	Reference
Combined Expansion	0.847 (0.763-0.940)<br>p=0.002	1.123 (0.971-1.300)<br>p=0.118

*Ratio of pre- to post- HR in non-expansion states compared to pre to post HR in combined expansion states. Ratios greater than 1 indicate more improvement in expansion states than in non-expansion states.

© 2020 Lam MB et al. *JAMA Network Open.*
